# Antitussive Activity of the Water-Extracted Carbohydrate Polymer from *Terminalia chebula* on Citric Acid-Induced Cough

**DOI:** 10.1155/2013/650134

**Published:** 2013-06-25

**Authors:** Gabriela Nosalova, Ludovit Jurecek, Udipta Ranjan Chatterjee, Sujay Kumar Majee, Slavomir Nosal, Bimalendu Ray

**Affiliations:** ^1^Department of Pharmacology, Jessenius Faculty of Medicine in Martin, Sklabinska 26, 036 01 Martin, Slovakia; ^2^Natural Products Laboratory, Department of Chemistry, The University of Burdwan, Burdwan, West Bengal 713 104, India; ^3^Clinic of Pediatric Anesthesiology and Intensive Medicine, Jessenius Faculty of Medicine and Martin University Hospital, Kollárova 2, 036 01 Martin, Slovakia

## Abstract

*Terminalia chebula*, a medicinal plant, is widely used in the management of various diseases. As the water extract of its dried ripe fruit is a frequently used preparation, we decided to look for bioactive polysaccharide in this extract. We demonstrate that the obtained polysaccharide fraction, CP, contained a highly branched arabinogalactan protein having a (1 → 3)-, (1 → 6)- and (1 → 3, 6)-linked **β**-D-Gal*p* together with (1 → 5)- and (1 → 3)-linked **α**-L-Ara*f* and nonreducing end units of **α**-L-Ara*f*. This polymer possesses strong antitussive property. Our results showed that the number of citric acid-induced cough efforts decreased significantly after the oral application of polysaccharide fraction in a dose of 50 mg kg^−1^ body weight. Its antitussive efficacy was higher than cough suppressive effect of standard drug codeine. Therefore, traditional aqueous extraction method provides a major polysaccharide, which induces a pharmacological effect: this could represent an attractive approach in phytotherapeutic managements.

## 1. Introduction

The cough reflex represents the most important defensive reflex of the airways, which together with mucociliary transport system forms the main mechanism for the cleaning of respiratory tract [1]. Coughing protects the breathing passages from blocking, thereby preventing the infected mucus from falling into lungs and bronchial tubes, which could be very dangerous. The cough reflex belongs to the most frequent symptoms of respiratory system diseases and is the most common reason why sick patients visit physicians [2, 3]. Pathological cough has significant impact on patient's quality of life observed either in physical activity or psychosocial domain. The present therapies are often limited for the lack of effective medications. Moreover, most of the existed medicines could bring about inevitable side effects. Thus, there is a need to develop new compounds with strong antitussive activity accompanied by favourable pharmacological and clinical properties. Natural products have been invaluable as biologically validated platforms for drug development [4–6]. Indeed, a series of review articles demonstrated the continuing and valuable contributions of nature as a source of lead compounds that have provided the basis and inspiration for the synthesis of new drugs [7, 8]. 

*Terminalia chebula*, a medicinal plant belonging to genus *Terminalia* (family, Combretaceae), is cultivated in Tibet, Taiwan, China, and India [9, 10]. The dried ripe fruit of *T. chebula*, locally known as haritaki, has been widely used for the treatment of fever, sore throat, cough, vomiting, hiccough, bleeding, piles, diarrhoea, gout, and heart and bladder diseases [11]. Haritaki fruits are reported to have free radical scavenging activities [12]. It is active against different gram-positive and gram-negative bacteria [13]. Phytochemical analysis of *T. chebula* shows the presence of gallic acid, chebulagic acid, corilagin, mannitol, ascorbic acid (vitamin C), and other compounds [14, 15]. The extract of *T. chebula* contained 24–64% tannins, 4.4% gallic acid, 22.2% total carbohydrate, and 5.3% uronic acid [15, 16]. Despite the general interest in phytoconstituents, it remains ironic that research on the chemical and biological aspects of the high molecular weight bioactive compounds has been neglected, although the aqueous extract of this fruit, which no doubt contains high molecular weight compound, is used in traditional medicine. Notably, many polysaccharides from medicinal plants exhibit a large range of pharmacological effects [17–20]. Hence, the aim of the present study was, first of all, to characterize the polysaccharide from *T. chebula* fruits. In addition, the antitussive activity of the isolated polysaccharide in terms of the number of cough efforts and specific airway resistance *in vivo* conditions in awaken male TRIK strain guinea pigs was evaluated. 

## 2. Materials and Methods

### 2.1. Plant Materials

Dry fruit powder (batch no. HTC008) of *Terminalia chebula* was purchased from Divya Pharmacy (Unit A-1, Haridwar, Uttarakhand, India). 

### 2.2. Chemicals

The chemicals used were of analytical grade or the best available. 

Codeine phosphate was purchased from Lachema (Brno, Czech Republic). Codeine phosphate, citric acid, and all samples were dissolved in water for injection. 

### 2.3. General Methods

Ultraviolet-visible (UV-VIS) spectra were recorded on a UV-24500 spectrophotometer (Shimadzu, Japan). IR spectra were obtained on a FT IR spectrophotometer (PerkinElmer FT/IR Spectrum RX 1) using a KBr discs containing finely ground samples. The ^1^H NMR spectrum of the water-extracted carbohydrate polymer (CP) was recorded on a Bruker 500 spectrometer (Bruker Biospin AG, Fallanden, Switzerland) operating at 500 MHz. These macromolecules were deuterium-exchanged by lyophilization with D_2_O (×3) and then examined as 1% solutions in D_2_O (99.96 atom % D). GC was carried out with a Shimadzu GC-17A chromatograph fitted with a flame ionization detector and a DB-225 column (30 m × 0.53 mm i.d.), using a program that maintained an isocratic temperature of 210°C for 18 min and helium as gas vector. GC-MS was performed with a Shimadzu QP 5050A GC-MS instrument at 70 eV. Conditions for GC-MS were as described previously [21]. Dialysis (molecular cutoff 12 kDa; Sigma-Aldrich) against distilled H_2_O were performed with continuous stirring. Evaporations were carried out under reduced pressure at around 50°C (SB 1100 Rotary Evaporator; Eyela, Tokyo, Japan), and small volumes of sample solutions were freeze-dried (Cool Safe 55-F freeze drier; Scanvac, Lynge, Denmark). Moisture content was determined by drying the ground material in an oven at 110°C for 3 h. Total carbohydrate content was estimated by the phenol-sulfuric acid assay [22] using glucose as standard. Total uronic acid was assayed as anhydrogalacturonic acid using *m*-hydroxydiphenyl color reagent [23]. Estimation of soluble protein content was performed by the Folin-Ciocalteu method. Amino acids were released by hydrolysis with 6 M HCl at 110°C for 22 h in a sealed tube and were analyzed as described previously [24]. Methylation was carried out by the method of Sims and Bacic [25]. In the methylation procedure, free hydroxyl groups in the carbohydrates were deprotonated and methylated, then the glycosidic linkages were hydrolyzed, and the partially methylated monosaccharides were reduced to alditols and acetylated. The partially methylated alditol acetates were analysed by GC and GC-MS using a DB-225 column (J&W Scientific) and using conditions as described previously [21]. The partially methylated alditol acetates were identified by the measurement of relative retention times, methoxyl substitution pattern as obtained from GC-MS, and carbohydrate composition of the nonmethylated polymers.

### 2.4. Isolation of Polysaccharide

Extraction of polysaccharide was conducted by stirring a suspension of *Terminalia chebula* fruit powder (20 g) in water (300 mL, pH 6.0) at 25–32°C for 12 h. Toluene was added to inhibit microbial growth. Separation of the residue from the extract was performed by filtration through a glass filter (G-2). The insoluble material was extracted twice more under similar condition at a solute to solvent ratio of 1 : 100 (w/v). The combined liquid extract was dialyzed extensively against water and lyophilized. The recovered material was dissolved in water, precipitated by the addition of ethanol (4 volumes), and then collected by centrifugation (×3). The final pellet was dissolved in water and lyophilized to yield the water-extracted carbohydrate polymer, named CP (0.563 g).

 The arabinogalactan protein (AGP) was isolated according to Schultz et al. [26]. Briefly, to a solution of CP in 1% NaCl (w/v) was added an equal volume of *β*-glucosyl Yariv reagent also in 1% NaCl. The mixture was kept at 4°C for 18 h and then centrifuged. The pellet was washed with 1% NaCl followed by pure methanol (×3), dried, and treated with sodium metabisulfite (10%). The resulting solution was dialyzed and freeze-dried to yield AGP.

### 2.5. Animals

Adult healthy awaken male TRIK strain guinea pigs, weighing 200–350 g, supplied by the department of experimental pharmacology, Slovak Academy of Science, Dobra Voda, Slovakia, were kept in faculty animal house with food and water *ad libitum* and with a standard air conditioning system. The animals were kept one week in quarantine before starting the experiment. 

The carbohydrate polymer (CP) from *T. chebula* fruit was tested on individual group of animals consisting of 20 guinea pigs, whereas the control agents (“positive” control codeine, “negative” control water for injection) were tested on 8 guinea pigs. All tested compounds were applied by peroral route of administration: carbohydrate polymer (CP) in the dose of 50 mg kg^−1^, codeine in the dose 10 mg kg^−1^, and water for injection in the dose 1 mL kg^−1^ body weight.

 The experimental protocols were approved by the Institutional Ethics Committee of the Jessenius Faculty of Medicine, Comenius University in Martin, Slovakia, registered in Institutional Review Board/Institutional Ethic Board Office (*IRB 00005636*), complied with Slovakian and European Community regulations for use of laboratory animals, and followed the criteria of experimental animal's wellfare. 

#### 2.5.1. Assessment of Chemically Induced Cough Reflex and Airways Defense Reflexes

Antitussive activity and reactivity of the airway smooth muscle* in vivo* were determined as described [27, 28]. Awaken guinea pigs were individually placed in a body plethysmograph box (HSE type 855, Hugo Sachs Elektronik, Germany) and restricted so that the head protrudes into the nasal chamber and the neck were sealed with a soft diaphragm. 

The cough reflex was induced by aerosol of citric acid in a concentration 0.3 M. The citric acid aerosol, generated by a jet nebulizer (PARI jet nebulizer, Paul Ritzau, Pari-Werk GmbH, Germany, output 5 L s^−1^, particles mass median diameter 1.2 *μ*m), was delivered to the head chamber of the plethysmograph for 3 min. The cough effort was defined as sudden PC-recorded enhancement of expiratory flow associated with typical cough motion and sound followed by two trained observers. Both influence on citric acid-induced cough and specific airway resistance were registered before any agent application (values labeled as N in graphs) and after that in 30, 60, 120, and 300 min time intervals. The minimal time interval between two measurements was 2 h for cough receptors adjustment as well as adaptation of laboratory animals on that kind of irritation [20, 29].

The reactivity of the airway smooth muscle *in vivo* conditions was expressed as values of specific airway resistance calculated according to Pennock et al. [30] by time difference between pressure changes in head and chest parts of body plethysmograph during normal breathing pattern. This noninvasive plethysmograph technique is commonly used for evaluation of bronchoactive substances effect [31]. The value of specific airway resistance is proportional to phase difference between nasal and thoracic respiratory airflow. The bigger the phase difference the higher is the value of specific airway resistance and hence, higher degree of bronchoconstriction. The values of sRaw were measured consecutively after the citric acid exposure and the cough response registration during 1 min interval. 

#### 2.5.2. Statistics

The changes of number of the citric acid-induced cough efforts and values of specific airways resistance in 30, 60, 120, and 300 min intervals were compared and statistically evaluated with initial data (*N* means number of cough efforts before application of agents). Student's *t*-test was used for the statistical analysis of the obtained results. Data are presented as mean ± standard error of the mean (SEM). *P* < 0.05 was considered statistically significant. Significance of *P* < 0.05, *P* < 0.01, and *P* < 0.001 is shown by one, two, or three asterisks, respectively. 

## 3. Results and Discussion 

In Indian ayurvedic system of medicine, a decoction of *Terminalia chebula* fruit powder in water is used as herbal remedy for chronic coughs and breathlessness [11]; therefore dry fruit powder of this medicinal plant was extracted with water. The yield of the water-extracted carbohydrate polymer (named as CP), after fractional precipitation with ethanol, was 29 mg per gram of dry fruit powder. Fraction CP contains 64% (w/w) neutral sugar along with 13% (w/w) protein. Aspartic acid (4 mol%), threonine (4 mol%), serine (5 mol%), glutamic acid (37 mol%), proline (9 mol%), glycine (10 mol%), alanine (6 mol%), cysteine (1 mol%), valine (3 mol%), isoleucine (2 mol%), leucine (5 mol%), tyrosine (1 mol%), phenylalanine (2 mol%), histidine (2 mol%), lysine (6 mol%), and arginine (3 mol%) were the amino acids associated with the protein present in fraction CP. The uronide content of this fraction was 2.3% (w/w). Sugar compositional analysis revealed that fraction CP consists mainly of arabinose, galactose, and glucose as the major neutral sugar in the molar ratio of 61 : 21 : 18. Therefore, this fraction may contain arabinogalactan. The high amount of glucose probably arises from starch, the presence of which has been confirmed by the blue coloration it developed with iodine reagent. Considering that the water extracted carbohydrate polymer (CP) contained galactosyl and arabinosyl residues as the major sugars and its protein content is 13%, we have tested its reactivity with *β*-glucosyl Yariv reagent that specifically precipitates arabinogalactan proteins (AGP). We found that the major part of CP was Yariv soluble. Sugar compositional analysis of this precipitate shows that it consisted mainly of galactose (77 mol%) residues and, to a lesser extent, arabinose (23 mol%) residues, confirming the presence of AGP. 

**Figure 1:**
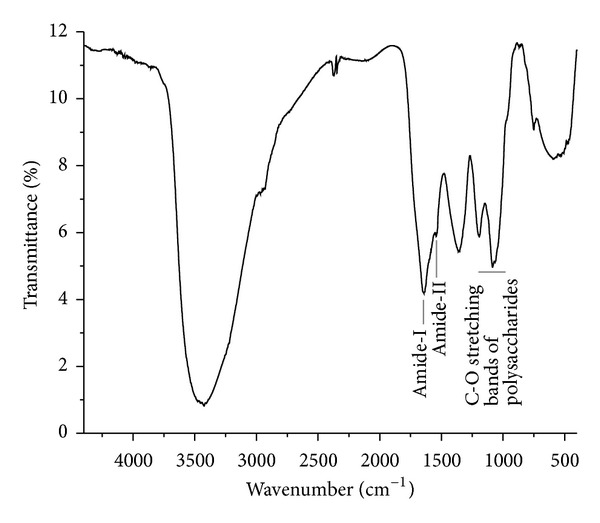
FT-IR spectrum of the carbohydrate polymer (CP) from *T. chebula*. The spectrum shows amide-I and amide-II bands at 1643 and 1542 cm^−1^ and carboxylate ion band at 1375 cm^−1^ together with bands at 1066 and 1098 cm^−1^ characteristics of carbohydrate.

We used FT-IR spectroscopy to assess the arabinogalactan protein structure. FT-IR spectroscopy is a vibrational spectroscopic method that can quantitatively detect, without derivatization, the relative amounts of functional groups, including –OH, NH_2_, amides, methyl, methylene, carbohydrate fingerprint region, with absorbance at certain wavenumber. FT-IR spectrum of the CP, as shown in Figure 1, contained bands characteristics of polysaccharide and protein. The stretching vibrations of –OH and –NH_2_ groups in sugar and protein moieties were indicated by a broad band around 3400 cm^−1^. Low-intensity bands around 2926 cm^−1^ derived from stretching resonances of CH, CH_2_, or CH_3_ groups in both monosaccharide and amino acid residues were also observed. It also contained bands at 1098 and 1066 cm^−1^ characteristic of carbohydrate. The presence of protein component was demonstrated by the occurrence of two bands of decreasing intensity: amide I towards 1643 cm^−1^ and amide II towards 1542 cm^−1^ with N–H and C–N links as reported previously for other proteins [32]. The chemical composition of CP and its IR spectrum suggests the presence of arabinogalactan protein. 

Methylation analysis showed that CP is a highly branched polysaccharide, containing nonreducing end units of Ara*f* (2,3,5-Me_3_-Ara) (16%), and Gal*p* (2,3,4,6-Me_4_-Gal) (4%). The arabinosyl units were substituted at *O*-5 (Ara*f*) and/or *O*-4 (Ara*p*) and *O*-3, in accord with 2,3-Me_2_-Ara (6%) and 2,5-Me_2_-Ara (4%) derivatives, respectively. The glucosyl units were substituted at *O*-4 and *O*-4,6, as demonstrated by the presence of 2,3,6-Me_3_-Glc (7%) and 2,3-Me_2_-Glc (1%) derivatives, respectively. The galactopyranosyl units are, mainly, 3-*O*- and 3,6-di-*O*-substituted, in accord with 2,4,6-Me_3_-Gal (19%) and 2,4-Me_2_-Gal (36%) methylated derivatives, respectively. Also present were 6-*O*-substituted galactopyranosyl units, as shown by the 2,3,4-Me_3_-Gal (7%) derivatives. Taken together, the glycosidic linkage composition of CP suggests the presence of arabinogalactan. 

**Figure 2:**
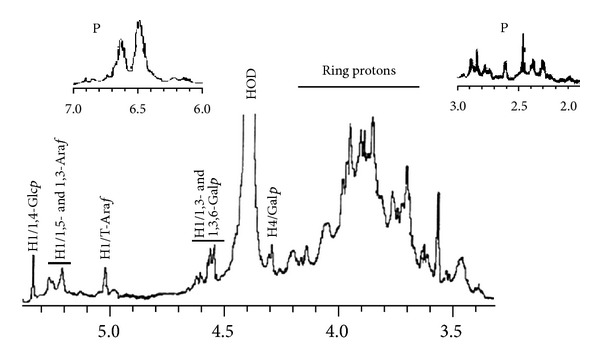
^1^H NMR spectrum at 500 MHz of the carbohydrate polymer (CP) from *T. chebula*. The spectrum of the macromolecule was recorded at 70°C in D_2_O solution. Insets: expansion of *δ*_*H*_ 6 to 7 and 2 to 3 regions. Numerical values are in *δ* (ppm). The signals for protein and deuterated water are designated as P and HOD, respectively.

The anomeric signals in the ^1^H NMR spectrum (Figure 2) of CP were assigned according to sugar composition, glycosidic linkage makeup, and data in the literature [33, 34]. The signal at **δ** 5.05 was assigned to anomeric protons (H1) of the nonreducing terminal **α**-Ara*f* and the signals in between **δ** 5.23 and **δ** 5.32 originated from the anomeric protons (H1) of 1,5- and 1,3-linked Ara*f*, respectively. This arabinogalactan also showed one group of H1 signals from *δ* 4.44 to 4.48 and a second group of H1 signals from *δ* 4.5 to 4.52 originating from resonances of anomeric protons (H1) of different **β**-Gal*p* residues of the **β**-1,3-linked galactan backbone. The signal at **δ**4.3 was assigned to H4 protons of **β**-Gal*p* residues. This spectrum also contained resonances characteristic of ring protons (H2–H5) between 3.5 and 4.2 ppm. The signal that appeared around 5.35 ppm can be attributed to anomeric proton of the Glc*p* moieties in CP. Signals for protein and deuterated water were designated as P and HOD, respectively. On the basis of the data obtained, it can be concluded that the carbohydrate polymer of *Terminalia chebula* fruit powder is an arabinogalactan protein.

**Figure 3:**
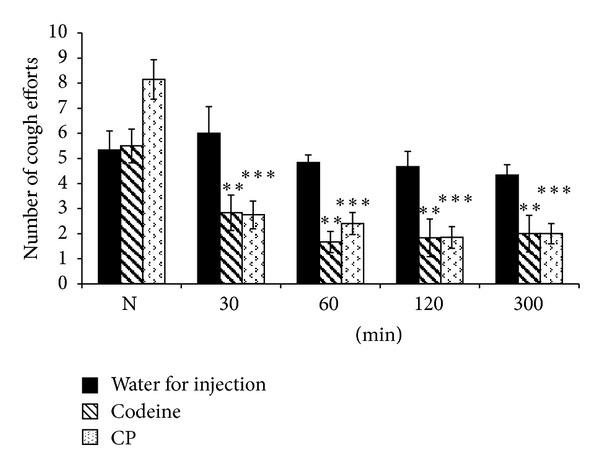
The influence of the carbohydrate polymer (CP) from *T. chebula*, codeine (positive control) and water for injection (negative control), on the citric acid-induced cough efforts in guinea pigs recorded at 30, 60, 120, and 300 min time intervals. The number of cough efforts before the application of the polysaccharides and codeine is given by *N*. *P* < 0.05 was considered statistically significant, and the significance of *P* < 0.05; *P* < 0.01; and *P* < 0.001 is shown by ∗, ∗∗, or ∗∗∗, respectively. All used substances were applied by peroral route of administration: plant polysaccharides in the dose of 50 mg kg^−1^, codeine in the dose 10 mg kg^−1^ and water in the dose 1 mL kg^−1^, body weight.

**Figure 4:**
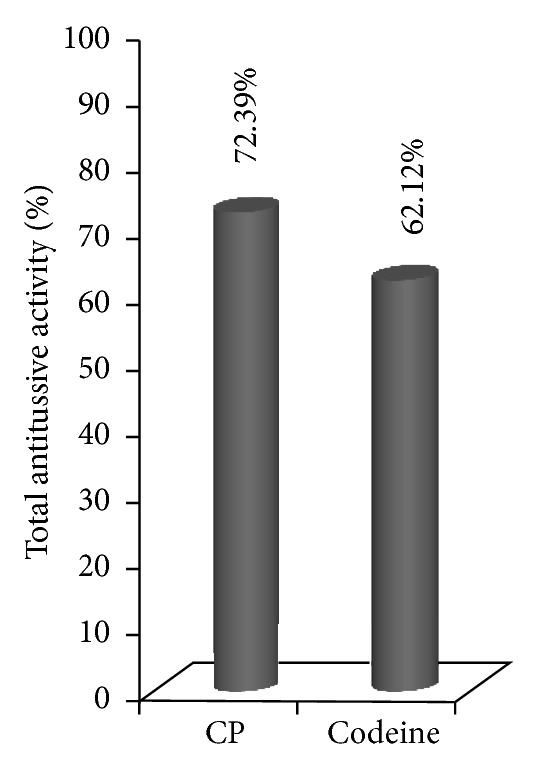
Total antitussive activity on guinea pigs of the carbohydrate polymer (CP) from *T. chebula* along with standard antitussive compound codeine (administered in the dose of 50 mg kg^−1^ and 10 mg kg^−1^ body weight, resp.).

The effect of polysaccharide component (CP) on the citric acid-induced cough reflex and reactivity of the airways smooth muscle using double chamber body plethysmograph were tested *in vivo* conditions. The cough reflex was chemically induced by exposing the guinea pigs to an aerosol of 0.3 M citric acid for 3 minutes. Cough efforts and specific airways resistances were registered before (N) and subsequently 30, 60, 120, and 300 minutes after oral administrations of CP as well as controls. The polysaccharide component CP was administered orally to 20 healthy male awaken guinea pigs in the dose of 50 mg kg^−1^ body weight. Efficiency of tested fraction was compared with antitussive activity of codeine (positive control) and water for injection (negative control). As shown in Figure 3, peroral administration of polysaccharide from *T. chebula* brought a highly significant (*P* < 0.001) decrease in number of cough efforts in the dose of 50 mg kg^−1^ body weight. This significant reduction of the cough efforts observed at 30 minutes after administration indicates prompt onset of action of the polysaccharide component. Furthermore, the positive effect on this parameter was observed during all the study time intervals. These results supported our previous finding that several naturally occurring polysaccharides possess antitussive activity [35–38]. Importantly, the total antitussive activity of CP was quantitatively higher than that of codeine, the strongest antitussive agent used in clinical practice (Figure 4). Narcotics such as codeine are not suitable for long-term treatment due to their habit-forming properties and unfavorable side effects including depressive activities on respiratory center and expectoration of mucus from the airways. These results showed not only the validity of the use of this plant in ayurvedic medicine but also the possibility to substitute codeine with more safer and effective antitussive agent from natural source.

**Figure 5:**
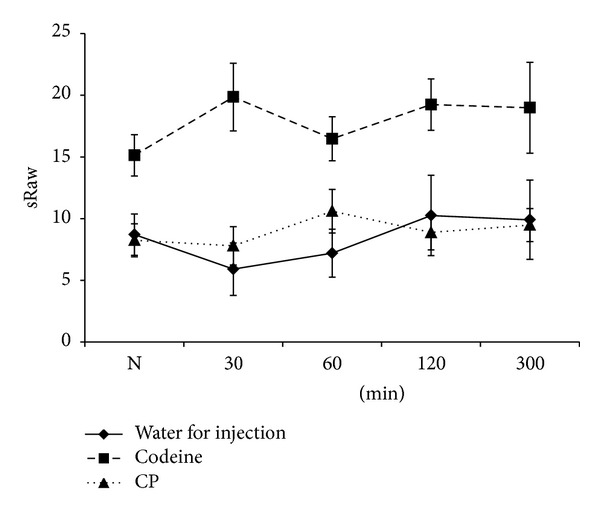
The influence of the carbohydrate polymer (CP) from the medicinal plant *T. chebula* and control agents (water for injection and codeine) on citric acid-induced changes of specific airway resistance (sRaw) *in vivo* conditions recorded at 0 (values labeled as *N* in graph), 30, 60, 120, and 300 min time intervals.

Airway resistance is a concept used in respiratory physiology to describe mechanical factors, which limit the access of inspired air to the pulmonary alveoli and thus determine airflow. It is dictated by, *inter alia*, the diameter of the airways. At present, the relationship between cough and bronchoconstriction is not known with certainty, although it is generally accepted that bronchodilating substances can cause cough suppression. Therefore, we have evaluated the changes of specific airway resistance as indictor of this activity. Our result suggests that the application of arabinogalactan from *T. chebula* in the dose, which provoked cough suppressive activities, did not significantly change the values of specific airway resistance (Figure 5). Therefore, this parameter do not modulates the antitussive activity of polysaccharide.

The mechanism(s) responsible for the antitussive activity of the polysaccharide from *T. chebula* cannot be defined from the present study. It may be due to spasmolytic activity, protective effects on mucous, and other mechanisms. Further studies in this area would be needed.

Arabinogalactan structures have been associated with various biological activities [18, 39–46]. Investigations of biological activities of arabinogalactan isolated from various medicinal plants emphasized the importance of highly branched side chains for the expression of the observed activities [18, 39–41, 45, 46]. Samuelsson and coworker [45] employing multivariate statistical analysis suggested that the magnitude of the biological activity of arabinogalactan is influenced by the content of certain side chains in the polymer. It has been shown that high activity correlates to large neutral side chains with high amounts of (1 → 6)- and (1 → 3,6)-linked Gal and low amounts of (1 → 4)-linked GalA. The arabinogalactan of the present study contains side chains possessing these distinctive features, and hence, these portions might be considered to be important as functional sites.

## 4. Conclusions

The findings of this study highlight several novel and important aspects of *T. chebula-*derived polysaccharide with regard to its structure and antitussive properties. A highly branched arabinogalactan protein could be isolated from the fruit powder of this medicinal plant using traditional water extraction protocol. This water-soluble macromolecule demonstrated strong antitussive activity. Moreover, administration of this compound did not change specific airways resistance significantly. The highly branched side chains of the arabinogalactan might be the functional sites. Finally, the biological activity observed in *T. chebula* provides a scientific basis for the use of the plant in traditional medicines.
